# Major Genes for Powdery Mildew Resistance in Research and Breeding of Barley: A Few Brief Narratives and Recommendations

**DOI:** 10.3390/plants14142091

**Published:** 2025-07-08

**Authors:** Antonín Dreiseitl

**Affiliations:** Department of Integrated Plant Protection, Agrotest Fyto Ltd., Havlíčkova 2787, CZ-767 01 Kroměříž, Czech Republic; dreiseitl@vukrom.cz

**Keywords:** *Blumeria hordei*, *Hordeum vulgare*, major resistance genes, gene postulation, specific resistance, boom-and-bust cycle, durable resistance

## Abstract

Genetic resistance is a sustainable way to protect crops from diseases, and breeding resistant varieties is a key objective. However, diseases are caused by pathogens with different life cycles, and the importance of individual evolutionary forces plays a key role in the adaptation of their populations. Therefore, strategies for the use of genetic resistance resources can vary depending on the plant pathosystem. Numerous major genes confer hypersensitive resistance to powdery mildew—one of the most common diseases in barley—but these genes conform to the gene-for-gene system of an extremely diverse and adaptable pathogen. When such resistance genes are transferred into commercial varieties, their efficiency in the field is soon overcome and replacement with newly developed resistant varieties can be slow. Hence, specific resistance genes should not be used in barley breeding programs. Only one monogenic, non-hypersensitive, non-specific and durable major resistance Mlo is known. This predominates in Central and Western European spring varieties and should be widely adopted by barley breeders elsewhere and in other crops where such type of resistance is found. In this paper, the relevant aspects involved in breeding barley resistant to powdery mildew are discussed, with conclusions supported by practical examples. Additionally, future directions for barley improvement are proposed.

## 1. Introduction

Breeding varieties resistant to harmful diseases is an important aim in crop breeding. However, pathogens have different life cycles and adaptation mechanisms (evolutionary forces), which are important in the promotion of population diversity and adaptability [1,2,3,4]. These aspects explain why strategies for crop protection, including the use of genetic resistance in different plant pathosystems, can differ. In the Czech Republic (Central Europe), the extensive study and practical use of major resistance genes in barley (*Hordeum vulgare* L.) against powdery mildew caused by *Blumeria hordei* (M. Liu & Hambl.) has resulted in increased knowledge, experience and conclusions [5,6,7,8,9,10,11,12,13,14], which are explored in this review. Despite the drawbacks of genes that provide specific resistance to barley powdery mildew, some research laboratories and breeding companies still use them in production of commercial varieties. This report outlines why this strategy should be modified in favor of adopting non-specific and durable resistance.

The co-evolution of barleys carrying specific resistances and local powdery mildew populations has created wide pathogen variation. This variation reflects specific resistance genes of the host in the pathogen population and can be utilized in postulating (identifying) of these resistance genes and their combinations. Additionally, knowledge of host variation can assist in detecting varietal non-authenticity and genotype heterogeneity, including gene bank accessions. Hence, pathotypes selected from populations of the pathogen that have overcome specific resistance genes and have resulted in losses in barley production can be used as a key tool for resistance gene postulation, thereby preventing losses through the use of non-authentic genotypes in research and breeding.

This study has three closely linked aims: (i) to present convincing examples of the rapid breakdown of race-specific resistance caused by the high adaptability of the pathogen, which leads to financial losses for growers, seed companies and breeders; (ii) to demonstrate how pathotypes that mostly arise from the breakdown of specific resistance can be used to postulate genes, helping to avert financial losses in barley research and breeding; and (iii) to recommend the adoption of durable resistance genes against powdery mildew for barley improvement.

## 2. Major Genes and Crop Resistance

Many major genes conferring disease resistance in crops have been detected. A common feature of genes selected for the breeding of resistant varieties is their efficiency against tested pathogen populations. Such genes provide resistance to varieties in the field, but usually for a limited time. Resistance genes are denoted as “major” because of their significant effectiveness against disease. The effectiveness of major specific resistance genes occurs in two dimensions, namely resistance to a disease when infected by a limited number of pathotypes or when infected by diverse pathogen populations.

### 2.1. Non-Durable Major Resistance Genes

**Figure 1:**
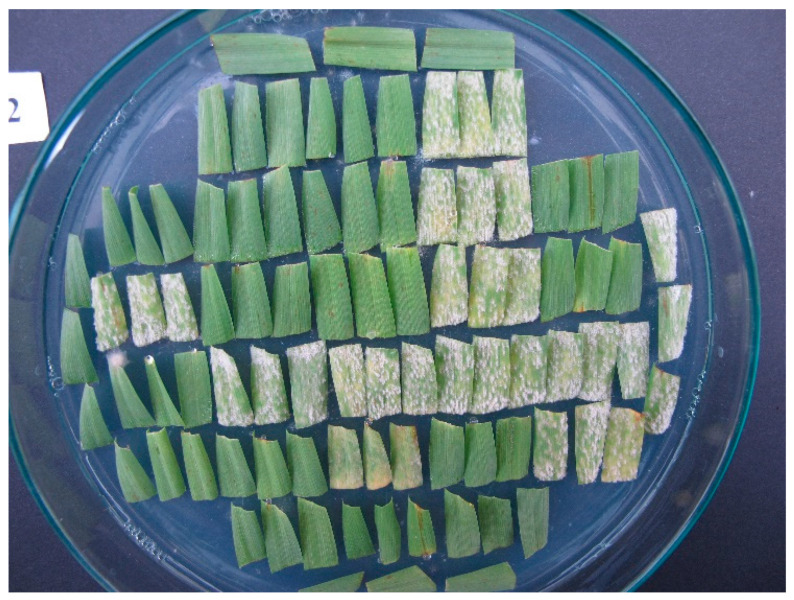
Petri dish with triplets of leaf segments from 30 barley lines, seven days after inoculation with the powdery mildew isolate Race I. Fully resistant (IR0) genotypes carry the *Mla8* allele.

An example of a highly efficient barley major resistance gene against powdery mildew is the allele *Mla8*, which is characterized by the lowest phenotype infection response 0 (IR0), meaning that no traces of the disease appear after the inoculation of the host genotypes with avirulent pathotypes (Figure 1). However, isolates that are avirulent to *Mla8* are extremely rare (see later) and absent in the field. Therefore, this gene is completely ineffective, and varieties containing *Mla8* are fully susceptible to “natural” pathogen populations worldwide.

Powdery mildew is a common disease wherever barley is grown. The pathogen has a mixed reproduction system that produces sexual and, more frequently, asexual spores. When a spore settles on a suitable host surface—mainly the leaves—a colony of the pathogen is formed, creating about 3 · 10^5^ new conidia in the field. The host has around 500–1000 fully developed tillers per square meter of canopy, and, on one tiller of an infected barley plant, many tens of colonies can occur. During the vegetation period of the crop, as well as later on volunteer plants and newly sown winter crops, many successive conidial generations are produced. This massive reproductive ability of the pathogen can result in large populations and explosive epidemics throughout the year. The spontaneous mutation frequency from avirulence to virulence has been estimated to be about 10^−8^ mutations per locus [2]; as a result, virulence against genes for specific resistance is continuously occurring. Conidia are spread by winds and can migrate up to hundreds of km [15], although the movement of spores steeply declines in the first few meters from mother colonies [16]. In addition to gene flow and mutations, the genetic variation in powdery mildew populations is also generated through sexual recombination. Chasmothecia are formed by fusion between two hyphae (ascogonium and antheridium) on adjacent colonies. Ascospores are formed in asci, and, when mature, they are discharged and dispersed by winds [2]. When new varieties possessing effective specific resistance genes or their combinations start to grow, the predominant part of the pathogen population (all avirulent pathotypes) is eliminated by this newly acquired resistance. This creates space for the unlimited reproduction of rare but diverse virulent pathotypes (i.e., directional selection), resulting in the rapid breakdown of specific resistance [3,17]. In Central Europe, this has led to one of the highest levels of population diversity observed among plant pathogens [12].

There are numerous well-characterized hypersensitive specific resistance genes against barley powdery mildew [18,19], and many others are present in wild barley (*H*. *vulgare* subsp. *spontaneum*) [20,21,22]. However, they conform to the principles of the gene-for-gene system, whereby each resistance gene of the host corresponds with a virulence gene of the pathogen [23], and their resistance is race-specific (denoted as “specific”). When used in commercial varieties, their initial resistance is soon overcome as a result of the increasing frequency of virulent pathotypes, which are under strong directional selection once varieties carrying such genes are grown [24]. The following examples illustrate the outcomes and conclusions drawn from the cultivation of varieties with major resistance genes.

In 1965, the domestic spring barley variety Diamant, an X-ray mutant from a superior variety Valticky, was registered. It possessed a new semi-dwarf sdw1.d allele [25]. Varieties bred from it are characterized as “Diamant (morpho)types”, and these semi-dwarf derivatives are often called the “Diamant series” (of varieties). Diamant has made a positive contribution to crop breeding worldwide but is susceptible to powdery mildew. This was the most common disease in cultivated barley in Europe, especially in the central and northwestern regions [2,26], until the expansion of spring varieties carrying a non-specific resistance gene. Diamant contains the ineffective resistance gene *Mla8*, and its average scoring in 161 field trials conducted in 1971–1975 was 3.77 on a nine-point scale, where 9 was the highest level of resistance [17]. The high susceptibility of Diamant encouraged breeders to combine the desirable agronomical characteristics of this variety with effective resistance to the disease.

**Figure 2:**
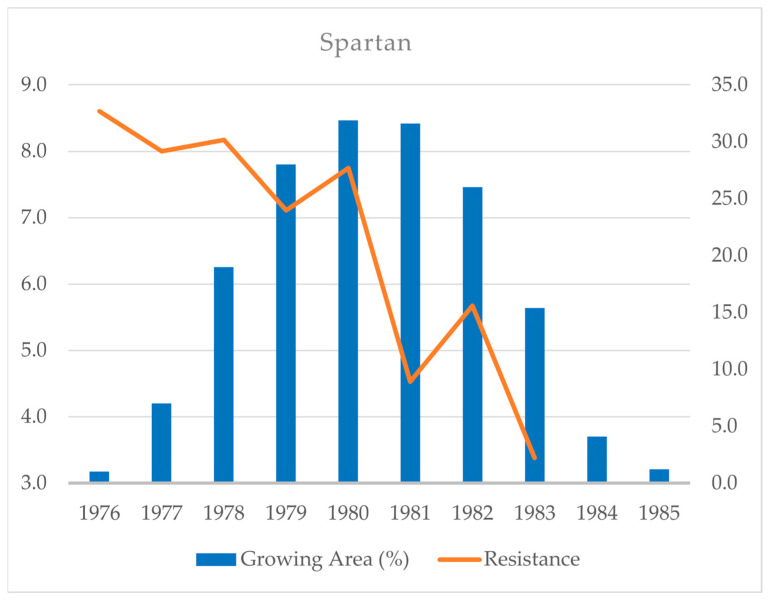
The resistance of Spartan, a spring barley variety carrying the resistance genes *Mla9* and *Mlk1* against powdery mildew, in the field (9 = full resistance) and its cultivation as a percentage of the total area of the crop grown in the Czech Republic in 1976–1985 [9,17].

In 1977, Spartan, a descendant of Diamant, was released, which carried a “new” gene, *Mla9,* as well as *Mlk1* [13], providing an overall resistance level of 8.60 in 39 field trials conducted a year before its registration [17]. Three years later, Spartan was historically the most widely grown variety in the Czech Republic, occupying 191,000 ha (31.9% of the crop area). The following year, when its area was 190,000 ha, the resistance was overcome (with an average field score of 4.53), and, in 1983, Spartan became the most susceptible (3.38) among all tested varieties. Since Spartan was the only commercial variety possessing *Mla9*, and because other resistant varieties were already available (see next paragraph), its growing area decreased quickly to 1.2% in 1985 (Figure 2). The widespread cultivation of Spartan and the progression of its susceptibility demonstrate a typical “boom-and-bust” cycle of varieties with specific resistance genes against diseases caused by pathogens that rapidly adapt [3].

**Figure 3:**
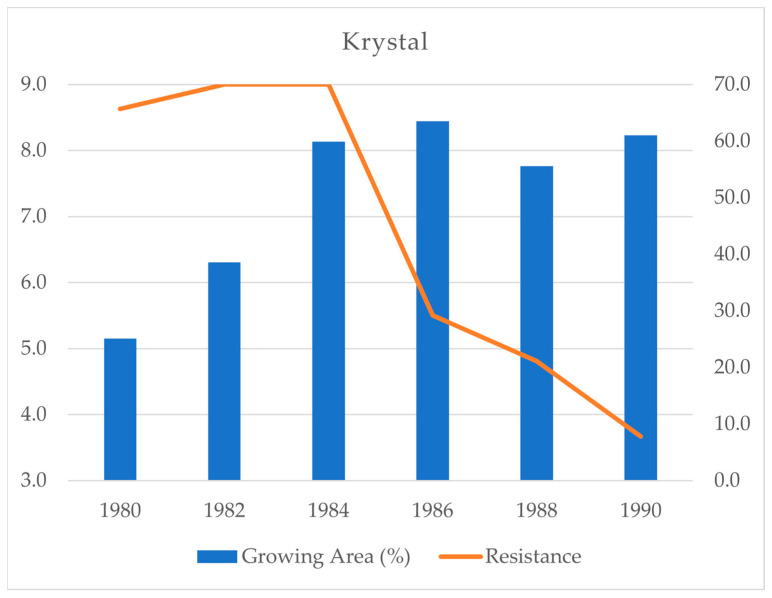
The resistance of Krystal, a spring barley variety carrying the resistance genes *Mla13* and *Mlg* against powdery mildew, in the field (9 = full resistance) and the area under cultivation as a percentage of varieties possessing *Mla13* grown in the Czech Republic from 1980 to 1990 [9,17].

Another example is the variety Koral, released in 1978, in which, similarly to Spartan, the *Mla8* allele from its ancestors was substituted with another allele—in this case *Mla13*, and simultaneously carrying *Mlg*. Identical resistance genes were also carried by Krystal, registered three years later [13]. Koral was the first Central European variety possessing *Mla13* [27], and its resistance averaged 8.93 in 105 field trials carried out in 1976–1978 [17]. Prior to 1985, five varieties with *Mla13* were registered in the Czech Republic, out of a total of 13 varieties registered in this eight-year period [13]. Varieties with *Mla13* were the most resistant for a decade (1976–1985) [17], and, up to 1985, they were grown on a total of about 1.5 Mha. After some infection in 1985, their resistance fully broke down in 1986, when these varieties occupied the largest proportion (63.5%) of the total crop area of 445,000 ha [9]. The years 1987–1989 had the fourth, first and second strongest mildew epidemics, respectively, that were recorded on spring barley over a 30-year period (1976–2005) [26], and, in 1989–2000, varieties carrying *Mla13* were the most susceptible in 8 of these 12 years [17]. Although the resistance was already ineffective, susceptible varieties possessing *Mla13* were still grown and occupied 61.0% of the crop area in 1990 (Figure 3) [9]. In addition, more varieties with *Mla13* were registered (10 until 1996 and three more up to 2011) because, until 1993, no other commercial resistant varieties were available [13,28]. The total area of *Mla13* varieties grown in 1986–2005, when they were fully susceptible, was 2.6 Mha, which was much larger than the area when they were resistant. The rapid spread of airborne spores of pathotypes containing important virulence genes (*Va13* and *Vg*, as well as *Va6*, *Va7*, *Va9* and *Vk1*) produced on these widely grown and already fully susceptible varieties caused significant powdery mildew epidemics in large parts of Europe in the second half of the 1980s [29].

In the domestic population of the pathogen, virulence against the resistance gene *Mlp* was not found until 2012, but, soon after the introduction of winter barley varieties carrying this gene, the frequency of corresponding virulence reached almost 70% [12]. The fate of varieties possessing other specific resistance genes, even those grown on small areas, was similar [17]. This raises questions about the benefit of specific resistance genes against barley powdery mildew. Despite these examples, and many other instances of specific resistance genes in barley rapidly losing their ability to control the disease, there are still research programs using this outdated strategy [30,31,32,33,34,35].

### 2.2. Durable Major Resistance Genes

Only one example of the durable, non-specific and non-hypersensitive major resistance of barley against powdery mildew that does not conform to the gene-for-gene concept is known [36,37]. It is monogenic, conditioned by one of many recessive genes (allels) of similar function [18], and designated as Mlo [38] (functional genes are designated *mlo*). In commercial varieties, *mlo11* derived from Ethiopian landraces was the first to be exploited. Subsequently, *mlo9* from a mutant SZ 5139 derived from Diamant was adopted by breeders, who released Alexis. This was the most widely grown European variety in the later part of the 1980s [37,39] (conversely, the powdery mildew super-susceptible line SM 4142 was selected from the same set of mutants and has been used in our laboratory).

Since 1993, 164 spring barley varieties, bred in nine Central and Northwestern European countries, have been registered in the Czech Republic, with 114 of them (69.5%) carrying Mlo resistance. During 2021–2023, almost 96% possessed Mlo, and, in registration trials, such varieties have been the most resistant since 1985 [24]. Despite its long-term durability, there is some experimental evidence that the pathogen can develop pathotypes that are partially virulent to Mlo [40,41,42,43]. There could be a higher risk of this occurring in areas where spring and winter barleys with this resistance are concurrently grown.

Jørgensen [37] predicted that Mlo resistance should also be present in other plants. This was confirmed when it was documented in some species in addition to barley [44]. With continuing research, the number of plant species with detected Mlo resistance is increasing [45,46,47,48,49,50,51,52,53,54].

### 2.3. Pathogen Variation

The successful postulation (see Section 2.4) of resistance genes in varieties depends on the use of the wide virulence variation of the pathogen, as outlined in the following examples.

The first European commercial varieties carrying introduced resistance genes were registered in the 1950s, since, prior to this date, no specific varietal resistance was detected. However, isolates that were avirulent to many varieties, including some of European origin, were found in Japan, and these isolates enabled the discovery of the gene *Mla8* [55]. In Denmark, a set of European varieties was tested with one of these Japanese isolates (Race I). From the results, it was concluded that *Mla8* was frequently present in old spring barley varieties [56]. In subsequently bred varieties, *Mla8* was almost always replaced with effective *Mla* alleles at the same locus [27], as in the cases of Spartan, Koral and Krystal mentioned above.

Another example relates to discussions held at the International Center for Agriculture Research in the Dry Areas (ICARDA) in 2004. Tadmor, a susceptible barley variety selected from a Syrian landrace Arabi Aswad [S. Ceccarelli, personal communication], was earmarked as a suitable host for powdery mildew experimental research. In resistance tests with about 50 isolates, Tadmor was fully susceptible (IR4) to all isolates except Race I, to which it was fully resistant (IR0), displaying an identical infection response array (IRA) to varieties possessing *Mla8*. However, in subsequent tests, a few Israeli isolates were included, and the set of barleys with IRAs previously indicating the presence of *Mla8* was split into two groups. Varieties in the first group had a similar IRA as before, when only Race I was avirulent (*Mla8*), whereas varieties in the other group, including Tadmor, were fully resistant (IR0) to this isolate, as well as to two Israeli isolates. Thus, the gene present in the second group, containing also winter varieties, differed from *Mla8*, and this newly discovered resistance was tentatively named after Tadmor (Ta). Despite this, a commercial winter barley variety Lomerit was present in the same group; later, it was considered more appropriate to designate the gene accordingly (*MlLo*). Subsequent research revealed that *MlLo* is an allele or pseudo-allele of the *Mla* locus [11,19]. Although *MlaLo* was undetected until recently, tests of gene bank accessions have shown that Lo is the most frequent resistance present alone or in combination with other powdery mildew resistances in more than 26% genotypes derived from old and current winter barley varieties [Dreiseitl and Nesvadba, unpublished].

Recent research has confirmed that the Central European barley powdery mildew population contains the widest spectrum of known virulence genes and their combinations among all regions where barley is grown and that it is one of most diverse populations among plant pathogens [12]. This is the cause of the short-term effectiveness of specific resistance genes and the main obstacle to their further use. On the other hand, selected isolates from this population are important in postulating the composition of complex resistance gene combinations, although the most frequent genes present in spring and winter barley (*Mla8* and *MlaLo*) could be revealed only by using non-European isolates because corresponding avirulence genes are absent in Europe.

### 2.4. Postulation of Major Resistance Genes

The classical identification of resistance genes in host varieties is based on the phenotypic responses of varieties after inoculation with pathogen isolates to obtain a row of IRs for each genotype. Comparing the IRAs of tested varieties with the IRAs of standard lines possessing known resistance genes can reveal genes and their combinations [6,13,32,57,58,59,60], including newly discovered resistance genes [61]. This method is denoted as the “postulation” of resistance genes [62] and is widely used to characterize genes in cereals against biotrophic pathogens, such as powdery mildews and rusts [63,64,65,66,67,68,69]. In the case of mildews, the term “postulation” was introduced later—first for mildew on wheat [70,71,72] and subsequently for barley mildew [10,73,74].

An integral step in exploiting resistance genes is their detection. Molecular markers [75,76,77,78,79] are helpful tools, mainly as an aid to select lines with the required gene(s). However, to distinguish new and more complex gene combinations, postulation is still the most suitable method, especially if maximum pathogen variation is used. Gene postulation based on a gene-for-gene concept in association with Mendelian genetic analysis was also used to clarify complex resistance genes in some accessions, including those that were highly heterogeneous [11]. The variation in host resistance to powdery mildew [18] was recently updated [19].

### 2.5. Other Uses of Major Resistance Genes

Plant research and breeding depend on access to a range of diverse plant genotypes that are available in gene banks. The non-authenticity of gene bank accessions due to human error is one of the most serious problems [10,80,81,82,83,84,85,86]. To overcome this shortcoming, there are modern and efficient methods of verifying the identity of plant varieties, including sequencing and protein spectra analyses [87,88]. However, these methods are often unsuitable for older accessions, such as those stored in gene banks, because of the lack of historical data to compare with current results obtained using modern techniques, and because present accessions can differ from original varieties due to frequent mislabeling or genotype contamination [89]. On the other hand, there is a lot of information about the presence of resistance genes against powdery mildew in barleys obtained at the time when varieties were registered or collected. Such data were summarized for almost 700 European varieties [27]. Therefore, the results of the current postulation of resistance genes in accessions, including those from gene banks, can be compared with original data, and, on this basis, varietal authenticity can be established. This problem is well illustrated in the following three examples resulting from the study of spring barley gene bank accessions.

For the resistance gene postulation of accessions maintained in a gene bank, five single-seed progenies (SSPs) of each accession were tested. The set of varieties included Abyssinian 1102, an Ethiopian landrace containing *mlo11*, one of the two most important recessive resistance alleles against barley powdery mildew and one of numerous sources of Mlo [37]. Three different genotypes were found among its SSPs, but none of them carried *mlo* [90]. This proves that this accession was heterogeneous and incorrectly labeled “Abyssinian 1102”.

Second, an accession of Diamant was studied [90]. Three lines (SSPs) were eliminated since they contained *mlo* and *Mla7*, both used in breeding barley varieties subsequent to Diamant [27]. Two SSPs carried *Mla8*, and only these can be considered as the true variety.

The last example is from a recent study comparing accessions from the domestic gene bank with those from foreign gene banks [89]. Asse (HOR 4482) is a German variety that is almost six decades old and contains *Mlg* [91]. In our studies, we found only *Mla8* in the USA accession and *Mlra* in the accession from the Czech gene bank. Because no true genotype was present in Asse accessions, a sample of this variety should be obtained from other gene banks, preferably from its country of origin.

These examples clearly demonstrate that knowledge of resistance genes present in varieties can be crucial. Even those genes whose resistance has broken down and are already ineffective are useful as “markers” or “information tools” in identifying varieties and confirming their pedigree, genotype homogeneity and authenticity.

## 3. Conclusions

The breeding of barley that is resistant to powdery mildew, especially in Europe, has been based almost exclusively on exploiting major resistance genes. Unlike the later-used non-specific resistance Mlo, specific resistance genes have a short-term protective effect that is terminated by the breakdown of resistance in the field and consequently associated with risks and financial losses for growers and breeding companies.

Other significant errors and financial losses occur when mislabeled or genetically heterogeneous varieties confound research aims or breeding projects and lead to erroneous scientific results and conclusions. This review presents examples of the first case (boom-and-bust cycle of varieties with specific resistance genes), summarizes the root causes and dangers using of non-authentic varieties that can be detected by identifying resistance genes against powdery mildew and highlights ways to avoid the associated losses.

## 4. Future Directions

Most of the known major resistance genes of barley against powdery mildew are race-specific and short-lived. The only exception is the non-specific resistance Mlo, which has remained durable after almost half a century of widespread use, mainly in Central and Western Europe, where the concurrent growing of spring and winter barley is commonly practiced and the conditions for the pathogen are favorable. In such an environment consisting of an uninterrupted green bridge, it is recommended that Mlo should be present only in spring varieties to prevent the slow, partial adaptation of the pathogen. For winter barley, it is advisable to accumulate quantitative (minor) non-specific resistance genes [92,93] or develop effective resistance introgressed from *Hordeum bulbosum* [94,95,96,97] or, if possible, from species in the tertiary gene pool of barley that are immune to closely related pathogens.

## Data Availability

All data are presented within the article.
